# The Benefits and Risks of Iron interventionS in Children (BRISC) trial: Statistical analysis plan

**DOI:** 10.12688/f1000research.23383.1

**Published:** 2020-05-22

**Authors:** Sabine Braat, Leila Larson, Julie A. Simpson, Mohammed Imrul Hasan, Jena Derakhshani Hamadani, Sheikh Jamal Hossain, Shamima Shiraji, Mohammad Saiful Alam Bhuiyan, Beverley-Ann Biggs, Sant-Rayn Pasricha

**Affiliations:** 1 Centre for Epidemiology and Biostatistics, University of Melbourne, Melbourne, Australia; 2 Department of Medicine and Victorian Infectious Diseases Service (Royal Melbourne Hospital), Peter Doherty Institute for Infection and Immunity, Melbourne, Australia; 3 Population Health and Immunity Division, The Walter and Eliza Hall Institute of Medical Research, Parkville, Australia; 4 Maternal and Child Health Division, International Centre for Diarrhoeal Disease Research, Dhaka, Bangladesh; 5 Department of Medical Biology, University of Melbourne, Melbourne, Australia; 6 Diagnostic Haematology at The Royal Melbourne Hospital, Clinical Haematology at The Royal Melbourne Hospital and Peter MacCallum Cancer Centre), Melbourne, Australia

**Keywords:** Iron, randomised control trial, child development, cognition, Bayley Scales, anaemia, statistical analysis plan, Bangladesh

## Abstract

**Background**: The Benefits and Risks of Iron interventionS in Children (BRISC) trial will evaluate the impact of universal supplementation with iron supplements or iron-containing multiple micronutrient powders (MNPs) compared with placebo given for 3 months on child development, growth, morbidity, laboratory indices of anaemia, iron deficiency, and inflammation at end of intervention and after a further 9 months post intervention in children aged 8 months living in rural Bangladesh. This paper describes the statistical analysis plan.

**Methods**: BRISC is a multi-site, three-arm, double-dummy blinded, parallel group, randomised control superiority trial in 3300 children. The statistical analysis plan was developed by the trial statistician in consultation with the trial steering committee and trial management committee based on the protocol, data collection forms, and study outcomes available in the blinded study database.

**Conclusion**: This detailed statistical analysis plan published prior to unblinding the allocated treatments will support the statistical analyses and reporting of the BRISC trial to be undertaken after unblinding. It allows for transparency as well as reproducibility of statistical analyses and reporting.

**Registration:** Australian New Zealand Clinical Trials Registry ACTRN12617000660381 (registered on 8 May 2017); World Health Organization Universal Trial Number U1111-1196-1125.

## Introduction

The World Health Organization (WHO) recommends daily iron supplementation to all children (universal provision) aged 6–23 months residing in settings where anaemia prevalence is 40% or above, or alternatively, home fortification with iron-containing multiple micronutrient powders where the prevalence of anaemia is 20% or above, with the goal of reducing anaemia and improving child development ^1, 2^. However, there is limited evidence for the effects of iron supplementation on early child development ^3, 4^; conversely, in high infection burden settings, iron may promote infection, including diarrhoea ^5^.

 The Benefits and Risks of Iron interventionS in Children (BRISC) trial is a placebo-controlled, randomised trial undertaken in rural Bangladesh designed to examine the effect of universal provision of iron syrup or iron-containing MNPs on child development, growth, morbidity from infections, and haematological and iron indices ^6^. The trial recruited the first participant in July 2017 and completed follow-up of the last participant in February 2020. The final results of the trial are expected to be submitted for publication by late 2020.

In this paper, the planned analyses for the BRISC trial are described. This plan supersedes the plan provided in the registry and published protocol ^6^. Finalisation of the statistical analysis plan prior to study unblinding has been undertaken to ensure transparency in the methods used to analyse and report the data and ultimately create the evidence for the effects of iron supplementation on early child development, growth, haemoglobin, iron status and infection.

## Methods

The trial protocol is summarised elsewhere ^6^.

### Aims

The primary objective of this study is to determine whether 3 months of iron supplementation or home fortification with MNPs is superior to placebo on cognitive development in 8-month old children at the end of the intervention. The secondary objectives are to evaluate the impact of iron supplementation and home fortification with MNPs, compared with placebo, on developmental indices, prevalence of anaemia and iron deficiency, growth, and infection risks at the end of the intervention and 9 months post-intervention.

### Design

**Figure 1.**
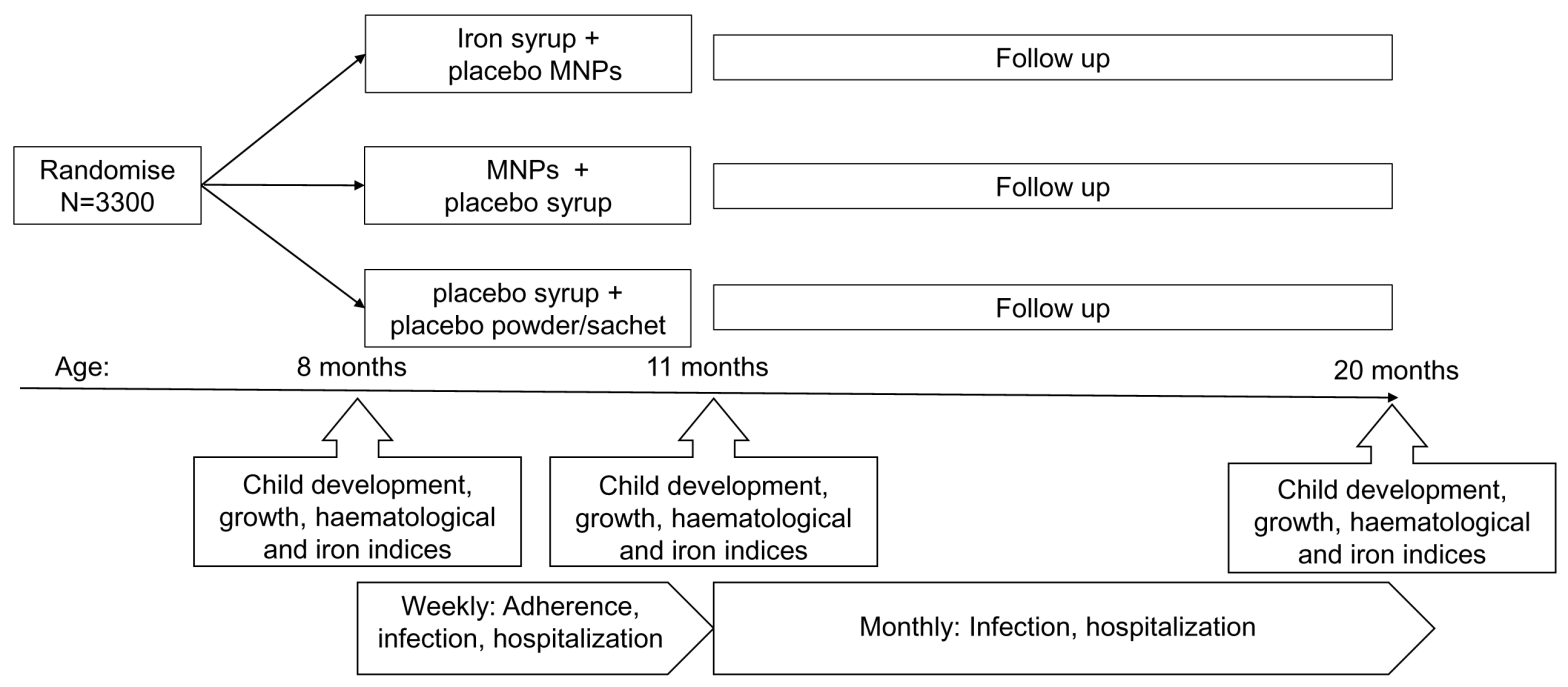
Study design.

BRISC is a three-arm, blinded, double-dummy, parallel group, placebo-controlled, individually-randomised, superiority trial. Starting at 8 months of age, children were randomised to either Arm 1: iron syrup (12.5 mg elemental iron) + placebo MNPs (powder sachet); Arm 2: MNPs (including 12.5 mg elemental iron) + placebo syrup; or Arm 3: placebo syrup and placebo powder/sachet (control), for 3 months ( Figure 1). Children were then followed up for an additional 9 months post-intervention. The study received ethics approval from the Ethical Review Committee of the International Centre for Diarrhoeal Disease Research, Bangladesh (icddr,b) and the Melbourne Health Human Research Ethics Committee (Melbourne, Australia). It was prospectively registered at the Australian New Zealand Clinical Trials Registry ( ACTRN1261700066038) and the World Health Organization (WHO) International Clinical Trials Registry Platform (U1111-1196-1125).

### Setting

The trial is conducted in Rupganj, a rural subdistrict/upazila of Narayanganj district about 50 km from Dhaka, in Bangladesh. Three unions (Bhulta, Golakandail, Rupganj) within the subdistrict are included, with each union covered by a dedicated field team.

### Participants

Children eligible for enrolment were children who were 8 months of age ±14 days, were not expected to leave the study site for more than 1 week over the next 3 months or for more than one month over the next 12 months and had a legally acceptable representative capable of providing consent. Children were excluded if they had severe anaemia (haemoglobin <80 g/L), if their drinking (ground) water iron content was >1 mg/L, if their mid upper arm circumference <11.5 cm, if they had been previously diagnosed with inherited red cell disorders, or if they had a current infective illness with fever.

### Randomisation and allocation

Children were randomly allocated to one of the three arms with 1:1:1 allocation using a computer-generated schedule of randomly permuted blocks of fixed size stratified by union and sex to achieve balance between the arms within each stratum. The randomisation list was computer-generated by an independent statistician. Blinding of the team visiting the site, the caregiver(s) and study participants was achieved through the use of identical packaging of sachets and syrup. Researchers, caregivers, persons involved with data collection (i.e., field team) or analysis will be blinded to the allocation code until the database has been cleaned and is ready for analysis.

### Outcome variables

All efficacy and laboratory outcomes were measured at baseline, 3 months post-intervention and after a further 9 months follow-up. Data related to infectious morbidity and hospitalisation were collected weekly during the intervention period and monthly during the follow-up period. Serious adverse events were measured at any time. The primary time-point of interest for all outcome variables is at the end of the intervention.

The primary outcome of the study is cognitive development, as measured using the cognitive composite score of the Bayley Scales of Infant and Toddler Development, Third Edition (Bayley III) ^7^. Bayley-III is a validated index of child development and the preferred field assessment tool. It is a standard series of measurements primarily to assess cognitive, motor (fine and gross) and language (receptive and expressive) development of infants and toddlers aged 0–3 ½ yrs. The total number of credited items is converted into scaled scores based on child’s age, which are then converted to composite scores of each subscale.

Key secondary outcomes include motor and language composite scores assessed by Bayley III, growth (length-for-age z-score, weight-for-age z-score), and laboratory indices (haemoglobin and ferritin concentrations). Secondary outcomes include other anthropometry measures (weight-for-length z-score, stunting, wasting, underweight, head circumference) and haematological and iron diagnoses (anaemia, iron deficiency, iron deficiency anaemia). Anthropometry outcomes (z-scores) will be derived using the child’s length and weight together with age and sex of the child according to age and sex specific WHO international reference growth standards ^8^. Using the z-scores, stunting will be defined as length-for-age z-score <-2, underweight as weight-for-age z-score <-2, and wasting as weight-for-length z-score <-2. Using the child’s haemoglobin (g/L), ferritin (µg/L), and C-reactive protein (mg/L), anaemia will be defined as haemoglobin <110 g/L, iron deficient as ferritin <12 µg/L or ferritin <30 µg/L if C-reactive protein >5 mg/L, and iron deficient anaemia as iron deficient and anaemia.

Exploratory outcomes include child’s behaviour using items from the Wolke’s Behaviour rating scale ^9^, consisting of nine behaviours each scored on a nine-point scale with higher scores indicating more favourable behaviour, and the temperament questionnaire ^10^, consisting of 33 questions each scored on a four-point scale with higher scores indicating a better temperament which will be grouped by summing into seven temperament summary scores.

Safety outcomes include infectious morbidity (includes fever, diarrhoea, bloody stool, vomiting, cough/ difficulty breathing), (serious) adverse events, and C-reactive protein (an inflammatory biomarker) and inflammation defined as C-reactive protein >5 mg/L.

Additional data collected included household baseline characteristics (union, religion, number of household members, parity, number of children under five years of age living in the household, maternal and paternal education, maternal and paternal occupation, wealth index ^11^, maternal depression ^12, 13^, household food insecurity ^14^) and child baseline characteristics (sex, age, currently breastfed, home stimulation as measured using the family care indicator score ^15^). Daily study medication intake was collected weekly during the 3-month intervention period and included reasons for non-adherence to the allocated treatment regimen. 

### Sample size

The sample size for the trial was to recruit 3300 children (1100 per treatment arm) in order to have 80% power to detect a two-point difference in Bayley III composite cognitive score between the iron supplementation and placebo arm and the MNPs and placebo arm (two-sided 2.5% level of significance per comparison), assuming a standard deviation of 15 and a 20% loss to follow up after 3 months of intervention. No interim analyses to stop the trial early were planned, and no interim analysis was conducted.

### Statistical analysis plan

The analysis will be conducted by statisticians from the University of Melbourne. After all study data are available and clean, a blinded data review meeting to review protocol violations, overall compliance, and missing data will be held prior to database lock. The final statistical analysis plan will be signed off during this meeting. The analysis of the primary outcome will be checked by an independent statistician. Discrepancies will be discussed and resolved by consensus.

### General principles

The intention-to-treat population will be used for the analysis of all primary, key secondary, secondary, and exploratory outcomes and will include all children who were randomised. In case of missing outcome data, we will follow the missing data handling strategy outlined below. The safety population will be used for the analysis of all safety outcomes and consists of all children who received at least one study treatment (including control). Children who have withdrawn informed consent for use of all their data will be excluded from all analyses. Children will be reported and analysed according to their randomised treatment allocation. Time-windows will be applied to all visit-wise data collected at baseline, month 3, and month 12, with assessments outside the predefined visit windows excluded from the analyses. Outcomes will be summarised using frequencies and percentages (based on the non-missing sample size) for categorical variables, mean and standard deviation for continuous variables, or median and quartiles (25th and 75th percentile) for non-symmetrical continuous variables. All analyses models will be adjusted for the stratification variables used during the randomisation (union and sex). All confidence intervals and P-Values will be two-sided.

### Trial profile

**Figure 2.**
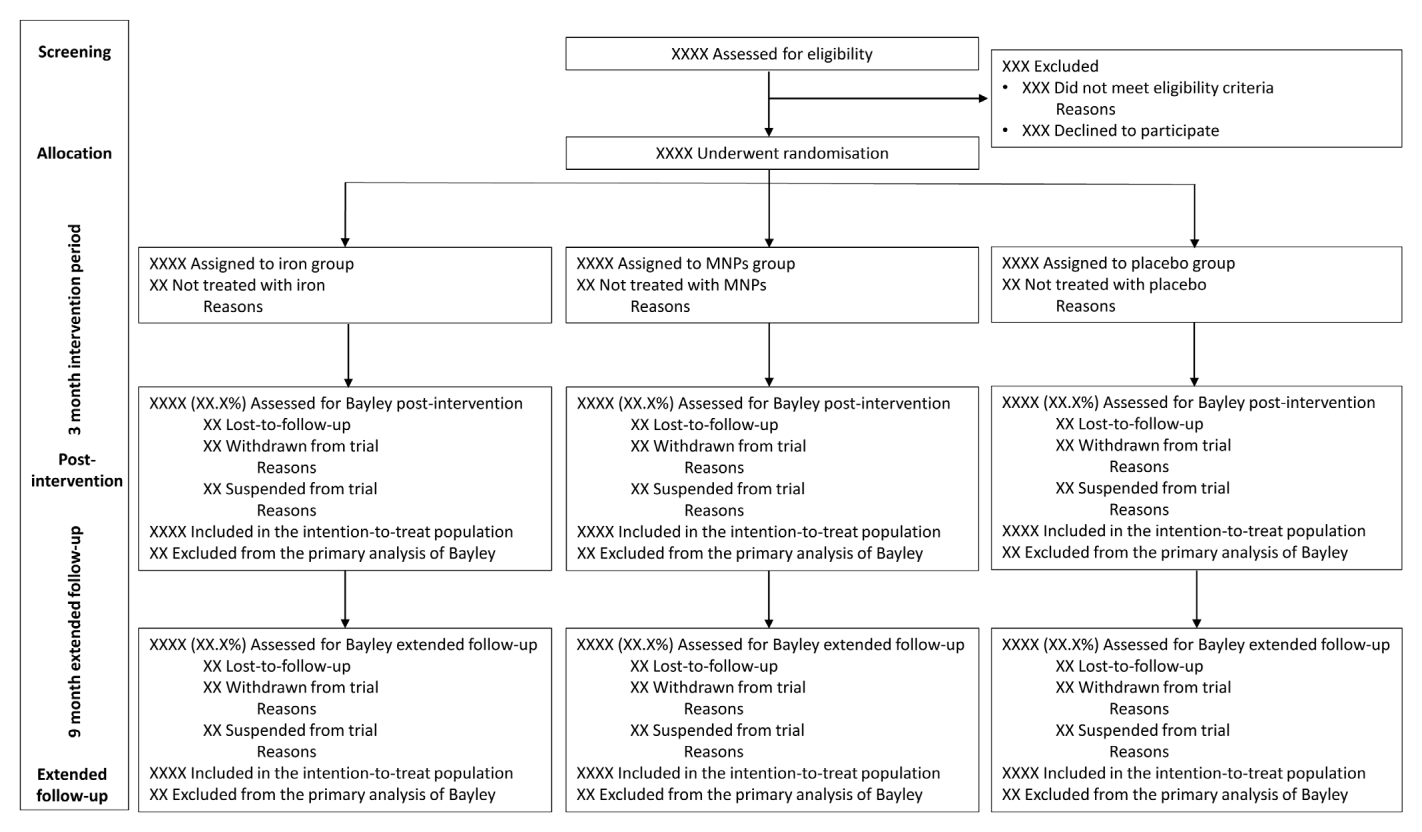
The CONSORT flow chart. MNPs denotes multiple micronutrient powders.

The flow of children through the trial will be presented in a Consolidated Standards of Reporting Trials (CONSORT) diagram, reasons for exclusion will be reported ( Figure 2).

### Baseline characteristics

Demographic and baseline variables of household and child characteristics will be summarised descriptively and presented by treatment group ( Table 1). No formal comparisons between groups will be made.

**Table 1.** Child and household characteristics at enrolment, according to treatment group (intention-to-treat population).

	Iron (N=XXXX)	MNPs (N=XXXX)	Placebo (N=XXXX)
**Household characteristic**			
Union - no. (%)			
Bhulta	XXX (XX.X)	XXX (XX.X)	XXX (XX.X)
Golakandail	XXX (XX.X)	XXX (XX.X)	XXX (XX.X)
Rupganj	XXX (XX.X)	XXX (XX.X)	XXX (XX.X)
Religion - no. (%)			
Islam	XXX (XX.X)	XXX (XX.X)	XXX (XX.X)
Other (Hindu, Buddhism, Christian)	XXX (XX.X)	XXX (XX.X)	XXX (XX.X)
Number of household members †	X (X-X)	X (X-X)	X (X-X)
Parity †	X (X-X)	X (X-X)	X (X-X)
Number of children under five years of age living in the household †	X (X-X)	X (X-X)	X (X-X)
Maternal education (years) †	X (X-X)	X (X-X)	X (X-X)
Paternal education (years) †	X (X-X)	X (X-X)	X (X-X)
Maternal occupation - no. (%)			
Unemployed	XXX (XX.X)	XXX (XX.X)	XXX (XX.X)
Skilled job	XXX (XX.X)	XXX (XX.X)	XXX (XX.X)
Unskilled job	XXX (XX.X)	XXX (XX.X)	XXX (XX.X)
Other	XXX (XX.X)	XXX (XX.X)	XXX (XX.X)
Paternal occupation - no. (%)			
Unemployed	XXX (XX.X)	XXX (XX.X)	XXX (XX.X)
Skilled job	XXX (XX.X)	XXX (XX.X)	XXX (XX.X)
Unskilled job	XXX (XX.X)	XXX (XX.X)	XXX (XX.X)
Other	XXX (XX.X)	XXX (XX.X)	XXX (XX.X)
Wealth index - no. (%)			
Quintile 1 (relative poorest)	XXX (XX.X)	XXX (XX.X)	XXX (XX.X)
Quintile 2	XXX (XX.X)	XXX (XX.X)	XXX (XX.X)
Quintile 3 (relative middle)	XXX (XX.X)	XXX (XX.X)	XXX (XX.X)
Quintile 4	XXX (XX.X)	XXX (XX.X)	XXX (XX.X)
Quintile 5 (relative wealthiest)	XXX (XX.X)	XXX (XX.X)	XXX (XX.X)
Maternal depression score †	X (X-X)	X (X-X)	X (X-X)
Household food insecurity score †	X (X-X)	X (X-X)	X (X-X)
**Child characteristic**			
** General**			
Female sex - no. (%)	XXXX (XX.X)	XXXX (XX.X)	XXXX (XX.X)
Age (months) *	X.X (X.X)	X.X (X.X)	X.X (X.X)
Currently breastfed - no. (%)	XXX (XX.X)	XXX (XX.X)	XXX (XX.X)
Family Care Indicator total score *	X.X (X.X)	X.X (X.X)	X.X (X.X)
** Laboratory indices**			
Haemoglobin concentration (g/L) *	XX.X (X.X)	XX.X (X.X)	XX.X (X.X)
Anaemia - no. (%)	XXX (XX.X)	XXX (XX.X)	XXX (XX.X)
Ferritin (ug/L) †	XX.X (XX.X-XX.X)	XX.X (XX.X-XX.X)	XX.X (XX.X-XX.X)
Iron deficient - no. (%)	XXX (XX.X)	XXX (XX.X)	XXX (XX.X)
Iron deficient anaemia - no. (%)	XXX (XX.X)	XXX (XX.X)	XXX (XX.X)
C-reactive protein (mg/L) †	XX.X (XX.X-XX.X)	XX.X (XX.X-XX.X)	XX.X (XX.X-XX.X)
Inflammation - no. (%)	XXX (XX.X)	XXX (XX.X)	XXX (XX.X)
** Child growth**			
Length-for-age z-score *	X.XX (X.XX)	X.XX (X.XX)	X.XX (X.XX)
Stunting - no.(%)	XXX (XX.X)	XXX (XX.X)	XXX (XX.X)
Weight-for-age z-score *	X.XX (X.XX)	X.XX (X.XX)	X.XX (X.XX)
Underweight - no. (%)	XXX (XX.X)	XXX (XX.X)	XXX (XX.X)
Weight-for-length z-score *	X.XX (X.XX)	X.XX (X.XX)	X.XX (X.XX)
Wasting - no. (%)	XXX (XX.X)	XXX (XX.X)	XXX (XX.X)
Head circumference (cm) *	X.XX (X.XX)	X.XX (X.XX)	X.XX (X.XX)
** Child development**			
Bayley score *			
Cognitive composite	XX.X (X.X)	XX.X (X.X)	XX.X (X.X)
Language composite	XX.X (X.X)	XX.X (X.X)	XX.X (X.X)
Motor composite	XX.X (X.X)	XX.X (X.X)	XX.X (X.X)
Wolke's behaviour score *			
Approach	X.X (X.X)	X.X (X.X)	X.X (X.X)
Adaptability	X.X (X.X)	X.X (X.X)	X.X (X.X)
General Emotional Tone	X.X (X.X)	X.X (X.X)	X.X (X.X)
Attentiveness	X.X (X.X)	X.X (X.X)	X.X (X.X)
Robustness	X.X (X.X)	X.X (X.X)	X.X (X.X)
Cooperation	X.X (X.X)	X.X (X.X)	X.X (X.X)
Vocalization	X.X (X.X)	X.X (X.X)	X.X (X.X)
Exploration	X.X (X.X)	X.X (X.X)	X.X (X.X)
Enthusiasm	X.X (X.X)	X.X (X.X)	X.X (X.X)
Temperament score			
Positive Emotionality *	XX.X (X.X)	XX.X (X.X)	XX.X (X.X)
Negative Emotionality *	X.X (X.X)	X.X (X.X)	X.X (X.X)
Fear *	X.X (X.X)	X.X (X.X)	X.X (X.X)
Social Approach *	X.X (X.X)	X.X (X.X)	X.X (X.X)
Orientation *	XX.X (X.X)	XX.X (X.X)	XX.X (X.X)
Related to Sleep †	XX.X (X.X-X.X)	XX.X (X.X-X.X)	XX.X (X.X-X.X)
Related to Energy and Exploration †	XX.X (X.X-X.X)	XX.X (X.X-X.X)	XX.X (X.X-X.X)

MNPs, multiple micronutrient powders. Percentages may not total 100 because of rounding. *Values are mean (SD). †Values are median (25th to 75th percentile).

### Multiple testing adjustment

The two primary comparisons of interest for the primary and key secondary outcomes are iron versus placebo and MNPs versus placebo. We will use a Bonferroni adjustment combined with a gatekeeping procedure to ensure control of the Type I error rate for the primary outcome across all three pairwise comparisons. Using the Bonferroni correction, we will test each of the two primary null hypotheses of no difference between iron and placebo and MNPs and placebo for the primary outcome at the two-sided 2.5% level of significance. If at least one of the two primary null hypotheses can be rejected (multiplicity unadjusted P-Value < 0.025), a comparison of iron versus MNPs will take place at either the two-sided 2.5% (if either iron or MNPs is superior to placebo) or 5% level of significance (if both iron and MNPs are superior to placebo). Estimates and two-sided confidence intervals will be presented with the same level of significance as used for the testing of these null hypotheses, along with multiplicity unadjusted and adjusted P-Values. If neither iron nor MNPs is superior to placebo, we will report the estimate and two-sided 95% confidence interval but not the P-Value for the comparison of iron versus MNPs.

For the set of key secondary outcomes, we will use the Hochberg procedure for each of the three pairwise comparisons at the level of significance used for the primary outcome only if the corresponding null hypothesis is rejected for the primary outcome. No multiplicity adjustment is planned for secondary and exploratory outcomes, and no P-Values will be presented.

We will follow the above outlined multiple testing approach for the primary and key secondary outcomes both for the primary time-point at month 3 and the secondary time-point at month 12 separately. The primary objective of the trial will have been met if one of the two primary null hypotheses for the primary outcome at the primary time-point can be rejected at the predetermined significance level. 

### Efficacy (including selected laboratory) outcomes: analysis

A constrained longitudinal data analysis method proposed by Liang and Zeger ^16^ will be used to examine the primary outcome (Bayley III cognitive composite score at baseline, 3 months and 12 months post-baseline). The model will incorporate study visit as a categorical variable, treatment and treatment by study visit interaction and adjust for the stratification randomisation factors (union and sex) as main effects. The model will assume a common baseline mean across the three treatment arms and an unstructured variance-covariance among the repeated measurements. In case of non-convergence, we will consider alternative structures (first-order autoregressive, Toeplitz, compound symmetry). The treatment effect will be estimated from this model as the difference between two treatments in mean change from baseline to 3 months post-intervention ( Table 2).

**Table 2.** Primary, key secondary, and secondary outcomes, according to treatment group (intention-to-treat population).

Assessment	Iron (N=XXXX)		MNPs (N=XXXX)		Placebo (N=XXXX)		Iron vs. Placebo			MNPs vs. Placebo			Iron vs. MNPs		
	no. of children measured	Mean (SD) *	no. of children measured	Mean (SD) *	no. of children measured	Mean (SD) *	Estimate (CI) †	P Value Unadjusted	P Value Adjusted †	Estimate (CI) †	P Value Unadjusted	P Value Adjusted †	Estimate (CI) †	P Value Unadjusted	P Value Adjusted †
Month 3															
Primary endpoint															
Bayley score															
Change in cognitive composite score	XXXX	X.X (X.X)	XXXX	X.X (X.X)	XXXX	X.X (X.X)	X.X (X.X to X.X)	0.XXX	0.XXX	X.X (X.X to X.X)	0.XXX	0.XXX	X.X (X.X to X.X)	0.XXX	0.XXX
Key secondary endpoints															
Bayley score															
Change in language composite score	XXXX	X.X (X.X)	XXXX	X.X (X.X)	XXXX	X.X (X.X)	X.X (X.X to X.X)	0.XXX	0.XXX	X.X (X.X to X.X)	0.XXX	0.XXX	X.X (X.X to X.X)	0.XXX	0.XXX
Change in motor composite score	XXXX	X.X (X.X)	XXXX	X.X (X.X)	XXXX	X.X (X.X)	X.X (X.X to X.X)	0.XXX	0.XXX	X.X (X.X to X.X)	0.XXX	0.XXX	X.X (X.X to X.X)	0.XXX	0.XXX
Anthropometric indices															
Change in length-for-age z-score	XXXX	X.X (X.X)	XXXX	X.X (X.X)	XXXX	X.X (X.X)	X.X (X.X to X.X)	0.XXX	0.XXX	X.X (X.X to X.X)	0.XXX	0.XXX	X.X (X.X to X.X)	0.XXX	0.XXX
Change in weight-for-age z-score	XXXX	X.X (X.X)	XXXX	X.X (X.X)	XXXX	X.X (X.X)	X.X (X.X to X.X)	0.XXX	0.XXX	X.X (X.X to X.X)	0.XXX	0.XXX	X.X (X.X to X.X)	0.XXX	0.XXX
Laboratory indices															
Change in haemoglobin concentration (g/L)	XXXX	X.X (X.X)	XXXX	X.X (X.X)	XXXX	X.X (X.X)	X.X (X.X to X.X)	0.XXX	0.XXX	X.X (X.X to X.X)	0.XXX	0.XXX	X.X (X.X to X.X)	0.XXX	0.XXX
Ferritin (µg/L)	XXXX	XX.X (XX.X- XX.X)	XXXX	XX.X (XX.X- XX.X)	XXXX	XX.X (XX.X- XX.X)	X.XX (X.XX to X.XX)	0.XXX	0.XXX	X.XX (X.XX to X.XX)	0.XXX	0.XXX	X.XX (X.XX to X.XX)	0.XXX	0.XXX
Secondary endpoints															
Anthropometric indices															
Stunting	XXXX	XX (X.X%)	XXXX	XX (X.X%)	XXXX	XX (X.X%)	X.XX (X.XX to X.XX)			X.XX (X.XX to X.XX)			X.XX (X.XX to X.XX)		
Underweight	XXXX	XX (X.X%)	XXXX	XX (X.X%)	XXXX	XX (X.X%)	X.XX (X.XX to X.XX)			X.XX (X.XX to X.XX)			X.XX (X.XX to X.XX)		
Change in weight-for- lenght z-score	XXXX	X.X (X.X)	XXXX	X.X (X.X)	XXXX	X.X (X.X)	X.X (X.X to X.X)			X.X (X.X to X.X)			X.X (X.X to X.X)		
Wasting	XXXX	XX (X.X%)	XXXX	XX (X.X%)	XXXX	XX (X.X%)	X.XX (X.XX to X.XX)			X.XX (X.XX to X.XX)			X.XX (X.XX to X.XX)		
Change in head circumference	XXXX	X.X (X.X)	XXXX	X.X (X.X)	XXXX	X.X (X.X)	X.X (X.X to X.X)			X.X (X.X to X.X)			X.X (X.X to X.X)		
Laboratory indices															
Anaemia	XXXX	XX (X.X%)	XXXX	XX (X.X%)	XXXX	XX (X.X%)	X.XX (X.XX to X.XX)			X.XX (X.XX to X.XX)			X.XX (X.XX to X.XX)		
Iron deficient	XXXX	XX (X.X%)	XXXX	XX (X.X%)	XXXX	XX (X.X%)	X.XX (X.XX to X.XX)			X.XX (X.XX to X.XX)			X.XX (X.XX to X.XX)		
Iron deficient anaemia	XXXX	XX (X.X%)	XXXX	XX (X.X%)	XXXX	XX (X.X%)	X.XX (X.XX to X.XX)			X.XX (X.XX to X.XX)			X.XX (X.XX to X.XX)		
Month 12															
Primary variable															
Change in cognitive composite score	XXXX	X.X (X.X)	XXXX	X.X (X.X)	XXXX	X.X (X.X)	X.X (X.X to X.X)	0.XXX	0.XXX	X.X (X.X to X.X)	0.XXX	0.XXX	X.X (X.X to X.X)	0.XXX	0.XXX
Key secondary variables															
Bayley score															
Change in language composite score	XXXX	X.X (X.X)	XXXX	X.X (X.X)	XXXX	X.X (X.X)	X.X (X.X to X.X)	0.XXX	0.XXX	X.X (X.X to X.X)	0.XXX	0.XXX	X.X (X.X to X.X)	0.XXX	0.XXX
Change in motor composite score	XXXX	X.X (X.X)	XXXX	X.X (X.X)	XXXX	X.X (X.X)	X.X (X.X to X.X)	0.XXX	0.XXX	X.X (X.X to X.X)	0.XXX	0.XXX	X.X (X.X to X.X)	0.XXX	0.XXX
Anthropometric indices															
Change in length-for-age z-score	XXXX	X.X (X.X)	XXXX	X.X (X.X)	XXXX	X.X (X.X)	X.X (X.X to X.X)	0.XXX	0.XXX	X.X (X.X to X.X)	0.XXX	0.XXX	X.X (X.X to X.X)	0.XXX	0.XXX
Change in weight-for-age z-score	XXXX	X.X (X.X)	XXXX	X.X (X.X)	XXXX	X.X (X.X)	X.X (X.X to X.X)	0.XXX	0.XXX	X.X (X.X to X.X)	0.XXX	0.XXX	X.X (X.X to X.X)	0.XXX	0.XXX
Laboratory indices															
Change in haemoglobin concentration (g/L)	XXXX	X.X (X.X)	XXXX	X.X (X.X)	XXXX	X.X (X.X)	X.X (X.X to X.X)	0.XXX	0.XXX	X.X (X.X to X.X)	0.XXX	0.XXX	X.X (X.X to X.X)	0.XXX	0.XXX
Ferritin (µg/L)	XXXX	XX.X (XX.X- XX.X)	XXXX	XX.X (XX.X- XX.X)	XXXX	XX.X (XX.X- XX.X)	X.XX (X.XX to X.XX)	0.XXX	0.XXX	X.XX (X.XX to X.XX)	0.XXX	0.XXX	X.XX (X.XX to X.XX)	0.XXX	0.XXX
Secondary variables															
Anthropometric indices															
Stunting	XXXX	XX (X.X%)	XXXX	XX (X.X%)	XXXX	XX (X.X%)	X.XX (X.XX to X.XX)			X.XX (X.XX to X.XX)			X.XX (X.XX to X.XX)		
Underweight	XXXX	XX (X.X%)	XXXX	XX (X.X%)	XXXX	XX (X.X%)	X.XX (X.XX to X.XX)			X.XX (X.XX to X.XX)			X.XX (X.XX to X.XX)		
Change in weight-for- lenght z-score	XXXX	X.X (X.X)	XXXX	X.X (X.X)	XXXX	X.X (X.X)	X.X (X.X to X.X)			X.X (X.X to X.X)			X.X (X.X to X.X)		
Wasting	XXXX	XX (X.X%)	XXXX	XX (X.X%)	XXXX	XX (X.X%)	X.XX (X.XX to X.XX)			X.XX (X.XX to X.XX)			X.XX (X.XX to X.XX)		
Change in head circumference	XXXX	X.X (X.X)	XXXX	X.X (X.X)	XXXX	X.X (X.X)	X.X (X.X to X.X)			X.X (X.X to X.X)			X.X (X.X to X.X)		
Laboratory indices															
Anaemia	XXXX	XX (X.X%)	XXXX	XX (X.X%)	XXXX	XX (X.X%)	X.XX (X.XX to X.XX)			X.XX (X.XX to X.XX)			X.XX (X.XX to X.XX)		
Iron deficient	XXXX	XX (X.X%)	XXXX	XX (X.X%)	XXXX	XX (X.X%)	X.XX (X.XX to X.XX)			X.XX (X.XX to X.XX)			X.XX (X.XX to X.XX)		
Iron deficient anaemia	XXXX	XX (X.X%)	XXXX	XX (X.X%)	XXXX	XX (X.X%)	X.XX (X.XX to X.XX)			X.XX (X.XX to X.XX)			X.XX (X.XX to X.XX)		

MNPs, multiple micronutrient powders; CI, confidence interval *Values are mean (SD) or no. (%), with the exception of ferritin which is median (25th to 75th percentile). †Multiplicity-adjusted according to the pre-specified multiple testing strategy.

Continuous key secondary, secondary, and exploratory outcomes will be analysed similarly as the primary outcome. Ferritin (µg/L) will be log _e_ transformed before analysis. Binary outcomes will be analysed using a generalised linear mixed model with a log-link function and binomial distribution, including child as a random intercept. In case of non-convergence, we will use a logit link function instead.

### Safety outcomes: analysis

The total number of times at least one infection (fever, diarrhoea, bloody stool, vomiting, cough/ difficulty breathing) was reported will be summarised per infection type during the intervention period (weekly reports), extended follow-up period (monthly reports), and (total) study period. The incidence rate ratio will be estimated using a Poisson regression model, with a logarithm of the time at risk as offset ( Table 3).

**Table 3.** Infectious morbidity, according to treatment group (safety population).

	Period	Iron *	MNPs *	Placebo *	Iron vs. Placebo		MNPs vs. Placebo		Iron vs. MNPs	
		(N=XXXX)	(N=XXXX)	(N=XXXX)	Ratio † (95% CI)	P Value	Ratio † (95% CI)	P Value	Ratio † (95% CI)	P Value
Fever - total number of times reported	Intervention period	XX (X-XX)	XX (X-XX)	XX (X-XX)	X.XX (X.XX to X.XX)	0.XXX	X.XX (X.XX to X.XX)	0.XXX	X.XX (X.XX to X.XX)	0.XXX
	Extended follow-up period	X (X-X)	X (X-X)	X (X-X)	X.XX (X.XX to X.XX)	0.XXX	X.XX (X.XX to X.XX)	0.XXX	X.XX (X.XX to X.XX)	0.XXX
	Study period	XX (X-XX)	XX (X-XX)	XX (X-XX)	X.XX (X.XX to X.XX)	0.XXX	X.XX (X.XX to X.XX)	0.XXX	X.XX (X.XX to X.XX)	0.XXX
Diarrhea - total number of times reported	Intervention period	XX (X-XX)	XX (X-XX)	XX (X-XX)	X.XX (X.XX to X.XX)	0.XXX	X.XX (X.XX to X.XX)	0.XXX	X.XX (X.XX to X.XX)	0.XXX
	Extended follow-up period	X (X-X)	X (X-X)	X (X-X)	X.XX (X.XX to X.XX)	0.XXX	X.XX (X.XX to X.XX)	0.XXX	X.XX (X.XX to X.XX)	0.XXX
	Study period	XX (X-XX)	XX (X-XX)	XX (X-XX)	X.XX (X.XX to X.XX)	0.XXX	X.XX (X.XX to X.XX)	0.XXX	X.XX (X.XX to X.XX)	0.XXX
Bloody stool - total number of times reported	Intervention period	XX (X-XX)	XX (X-XX)	XX (X-XX)	X.XX (X.XX to X.XX)	0.XXX	X.XX (X.XX to X.XX)	0.XXX	X.XX (X.XX to X.XX)	0.XXX
	Extended follow-up period	X (X-X)	X (X-X)	X (X-X)	X.XX (X.XX to X.XX)	0.XXX	X.XX (X.XX to X.XX)	0.XXX	X.XX (X.XX to X.XX)	0.XXX
	Study period	XX (X-XX)	XX (X-XX)	XX (X-XX)	X.XX (X.XX to X.XX)	0.XXX	X.XX (X.XX to X.XX)	0.XXX	X.XX (X.XX to X.XX)	0.XXX
Vomiting - total number of times reported	Intervention period	XX (X-XX)	XX (X-XX)	XX (X-XX)	X.XX (X.XX to X.XX)	0.XXX	X.XX (X.XX to X.XX)	0.XXX	X.XX (X.XX to X.XX)	0.XXX
	Extended follow-up period	X (X-X)	X (X-X)	X (X-X)	X.XX (X.XX to X.XX)	0.XXX	X.XX (X.XX to X.XX)	0.XXX	X.XX (X.XX to X.XX)	0.XXX
	Study period	XX (X-XX)	XX (X-XX)	XX (X-XX)	X.XX (X.XX to X.XX)	0.XXX	X.XX (X.XX to X.XX)	0.XXX	X.XX (X.XX to X.XX)	0.XXX
Cough/Difficulty breathing - total number of times reported	Intervention period	XX (X-XX)	XX (X-XX)	XX (X-XX)	X.XX (X.XX to X.XX)	0.XXX	X.XX (X.XX to X.XX)	0.XXX	X.XX (X.XX to X.XX)	0.XXX
	Extended follow-up period	X (X-X)	X (X-X)	X (X-X)	X.XX (X.XX to X.XX)	0.XXX	X.XX (X.XX to X.XX)	0.XXX	X.XX (X.XX to X.XX)	0.XXX
	Study period	XX (X-XX)	XX (X-XX)	XX (X-XX)	X.XX (X.XX to X.XX)	0.XXX	X.XX (X.XX to X.XX)	0.XXX	X.XX (X.XX to X.XX)	0.XXX

MNPs, multiple micronutrient powders; CI, confidence interval Intervention period – 0–3 months, Extended follow-up period 4–12 months, Study period 0–12 months. *Values are median (25th to 75th percentile). †Ratio is incidence rate ratio.

The number and percentage of children who died, had at least one (overnight) hospitalisation, reported at least one serious adverse event, and had at least one clinic visit (due to any infection, fever, diarrhoea, bloody stool, vomiting, cough/ difficulty breathing, other infection) during the intervention period, extended follow-up period, and study period will be reported and compared between treatments using a log-binomial regression model ( Table 4). 

**Table 4.** Death, serious adverse events, hospitalisation, according to treatment group (safety population).

	Period		Iron (N=XXXX)	MNPs (N=XXXX)	Placebo (N=XXXX)	P-Value Iron vs. Placebo	P-Value MNPs vs. Placebo	P-Value Iron vs. MNPs
Children who died - no. (%)								
	Intervention period		XXX (XX.X)	XXX (XX.X)	XXX (XX.X)	0.XXX	0.XXX	0.XXX
	Extented follow-up period		XXX (XX.X)	XXX (XX.X)	XXX (XX.X)	0.XXX	0.XXX	0.XXX
	Study period		XXX (XX.X)	XXX (XX.X)	XXX (XX.X)	0.XXX	0.XXX	0.XXX
Children ≥1 hospitalisation * - no. (%)								
	Intervention period		XXX (XX.X)	XXX (XX.X)	XXX (XX.X)	0.XXX	0.XXX	0.XXX
	Extended follow-up period		XXX (XX.X)	XXX (XX.X)	XXX (XX.X)	0.XXX	0.XXX	0.XXX
	Study period		XXX (XX.X)	XXX (XX.X)	XXX (XX.X)	0.XXX	0.XXX	0.XXX
Children with ≥1 SAE - no. (%)								
	Intervention period		XXX (XX.X)	XXX (XX.X)	XXX (XX.X)	0.XXX	0.XXX	0.XXX
	Extented follow-up period		XXX (XX.X)	XXX (XX.X)	XXX (XX.X)	0.XXX	0.XXX	0.XXX
	Study period		XXX (XX.X)	XXX (XX.X)	XXX (XX.X)	0.XXX	0.XXX	0.XXX
Children ≥1 clinic visit † - no. (%)								
	Intervention period							
		Any reason	XXX (XX.X)	XXX (XX.X)	XXX (XX.X)	0.XXX	0.XXX	0.XXX
		Due to fever	XXX (XX.X)	XXX (XX.X)	XXX (XX.X)	0.XXX	0.XXX	0.XXX
		Due to diarrhea	XXX (XX.X)	XXX (XX.X)	XXX (XX.X)	0.XXX	0.XXX	0.XXX
		Due to bloody stool	XXX (XX.X)	XXX (XX.X)	XXX (XX.X)	0.XXX	0.XXX	0.XXX
		Due to vomiting	XXX (XX.X)	XXX (XX.X)	XXX (XX.X)	0.XXX	0.XXX	0.XXX
		Due to cough/ difficulty breathing	XXX (XX.X)	XXX (XX.X)	XXX (XX.X)	0.XXX	0.XXX	0.XXX
		Due to other ‡	XXX (XX.X)	XXX (XX.X)	XXX (XX.X)	0.XXX	0.XXX	0.XXX
	Extended follow-up period							
		Any reason	XXX (XX.X)	XXX (XX.X)	XXX (XX.X)	0.XXX	0.XXX	0.XXX
		Due to fever	XXX (XX.X)	XXX (XX.X)	XXX (XX.X)	0.XXX	0.XXX	0.XXX
		Due to diarrhea	XXX (XX.X)	XXX (XX.X)	XXX (XX.X)	0.XXX	0.XXX	0.XXX
		Due to bloody stool	XXX (XX.X)	XXX (XX.X)	XXX (XX.X)	0.XXX	0.XXX	0.XXX
		Due to vomiting	XXX (XX.X)	XXX (XX.X)	XXX (XX.X)	0.XXX	0.XXX	0.XXX
		Due to cough/ difficulty breathing	XXX (XX.X)	XXX (XX.X)	XXX (XX.X)	0.XXX	0.XXX	0.XXX
		Due to other ‡	XXX (XX.X)	XXX (XX.X)	XXX (XX.X)	0.XXX	0.XXX	0.XXX
	Study period							
		Any reason	XXX (XX.X)	XXX (XX.X)	XXX (XX.X)	0.XXX	0.XXX	0.XXX
		Due to fever	XXX (XX.X)	XXX (XX.X)	XXX (XX.X)	0.XXX	0.XXX	0.XXX
		Due to diarrhea	XXX (XX.X)	XXX (XX.X)	XXX (XX.X)	0.XXX	0.XXX	0.XXX
		Due to bloody stool	XXX (XX.X)	XXX (XX.X)	XXX (XX.X)	0.XXX	0.XXX	0.XXX
		Due to vomiting	XXX (XX.X)	XXX (XX.X)	XXX (XX.X)	0.XXX	0.XXX	0.XXX
		Due to cough/ difficulty breathing	XXX (XX.X)	XXX (XX.X)	XXX (XX.X)	0.XXX	0.XXX	0.XXX
		Due to other ‡	XXX (XX.X)	XXX (XX.X)	XXX (XX.X)	0.XXX	0.XXX	0.XXX

MNPs, multiple micronutrient powders; SAE, serious adverse event. Intervention period – 0–3 months, Extended follow-up period 4–12 months, Study period 0–12 months. *Hospitalisation is defined as an overnight stay. †Clinic visit is defined as visit to the clinic not resulting in hospitalisation. ‡Other is defined as stool with mucous, runny nose, skin problem, eye problem, oral problem, ear problem, constipation, check up, other.

C-reactive protein levels will be analysed using similar models as those described for the primary outcome and for inflammation similar models to those described for the binary key secondary and secondary outcomes.

We will present the multiplicity unadjusted P-Values for the safety outcomes, no multiple testing adjustment is planned.

### Missing data handling

To describe the missing data, the frequency and percentage of children with missing data at baseline, month 3 and month 12 will be summarised for the child development, anthropometry, and laboratory outcomes. In addition, baseline and demographic characteristics will be summarized by those with and without missing data for the cognitive composite score (at baseline, month 3, and month 12) to explore the missing data assumption and identify any variables not included in the target analyses that are potentially associated with missingness (known as auxiliary variables).

As the primary strategy to handle missing data, the analysis will use a likelihood-based approach. This approach relies on the underlying assumption that the probability of missing outcome data is not related to the missing data but to some of the observed measured data in the model (Missing At Random [MAR]). 

As the secondary strategy (sensitivity analysis), missing data on the outcomes will be multiply imputed using chained equations. The imputation model will include union, sex, visit (categorical), the family care indicator total score (continuous), maternal education (continuous), all variables listed as specified for subgroup analyses, and it will be performed separately by treatment group. In addition, auxiliary variables identified during the blinded data review meeting may be included. The variables with missing data will be imputed using a linear regression model if continuous and logistic regression if binary, whereby ferritin will be log _e_ transformed prior to imputation as this outcome will be log _e_ transformed in the analysis of interest. The missing outcome data at baseline, 3-month and 12-month visits will be imputed using the “just another variable” approach (also known as imputing in wide format) which requires a separate imputation model for imputing the variable at each assessment time. The number of imputed data sets will be greater than or equal to the percentage of missing data in the available case analyses. The imputed data sets will be analysed using the models described. The estimates from the analyses of the imputed data sets will be combined to obtain a pooled common estimate and corresponding confidence interval using Rubin’s rules. For the above standard implementation of multiple imputation, we have assumed the outcome data are MAR.

### Adherence

Overall compliance across the 3-month intervention period will be derived as the total number of days the child has reported taking both the syrup and the sachet divided by the child’s study participation duration, with ‘complier’ defined as those with overall compliance ≥70%. If no data on treatment intake is available, compliance will be assumed to be 0%.

### Sensitivity analyses

In addition to the analyses specified for the primary, key secondary, and secondary outcomes, the following sensitivity analyses will be applied for these outcomes:

1. Analyses consisting of models adjusted for potential prognostic or predictive variables:

a. Adding to the model adjusted for union and sex, the main effect of family care indicator score (continuous) and maternal education (No education; 1–8 years schooling completed; 9–12 years schooling completed; >12 years schooling completed).
b. Adding to the model adjusted for union and sex, the main effect of variables in Table 1 demonstrating unexpected imbalance between the treatment arms after unblinding.
c. Adding to the model adjusted for union and sex, the main effect for rater and the interaction between rater and study visit (only applies to Bayley III, Wolke’s Behaviour rating scale, and temperament questionnaire).
d. Adding to the model adjusted for union, sex, family care indicator, and maternal education, the main effect for rater and the interaction between rater and study visit (only applies to Bayley III, Wolke’s Behaviour rating scale, and temperament questionnaire).

2. Analyses of the secondary strategy to handle missing data.

3. Analyses of the model adjusted for union and sex for the per-protocol population defined as randomised children who were compliant to treatment, and without protocol violations (no informed consent or withdrawn informed consent for use of all data, violation in/ exclusion criteria, or improper unblinding of the child’s allocated treatment).

4. Analyses using the Complier Average Causal Effect method to estimate the average effect of treatment among compliers ^17^.

### Subgroup analyses

Exploratory subgroup analyses will be performed for cognitive motor and language composite scores assessed by Bayley III at 3 and 12 months. Subgroup (main effect) and the subgroup-by-treatment-by-visit interaction (as well as subgroup-by-treatment and subgroup-by-visit interaction) terms will be added to the constrained longitudinal data analysis model to evaluate whether the treatment effect differs between subgroup categories. The following subgroups will be explored: sex (male/female), baseline anaemia status (yes/no), baseline iron deficient status (yes/no), baseline iron deficient anaemia status (yes/no), baseline stunting (yes/no), baseline home stimulation as measured by the family care indicator questionnaire (below/ above median family care indicator total score), baseline household food insecurity status (yes/no), baseline wealth status (below/ above median wealth index score), and union (Bhulta, Golakandail, Rupganj). No multiplicity adjustments are planned for the subgroup analyses due to their explorative nature, we will present the estimates and two-sided 95% confidence interval along with (multiplicity unadjusted) P-Values. Results of the subgroup analyses will be displayed using Forest plots.

### Changes from the registry and published study protocol

This paper includes changes to the statistical analysis plan of the BRISC trial in the registry (registered May 2017) and protocol paper (accepted September 2017). These changes are:

- The register states that the regression models will incorporate key confounders and unbalanced baseline factors into the model. Instead, the primary analysis model will include the stratification factors used during the randomisation and additional models (sensitivity analyses) will incorporate key confounders, or key confounders and unbalanced baseline factors.
- The protocol states on the topic of:
  - Multiple testing: We detailed a multiple testing procedure for the primary and key secondary outcomes, thus the confidence level for the associated two-sided confidence interval will be less than 5% for some treatment effects.
  - Analysis of binary outcomes: We changed the analysis model from generalised estimating equations to a mixed model.

This paper documents version 1 of the statistical analysis plan dated April 20, 2020. Any changes to this version between publishing and breaking of the code will be tracked and still considered as planned analyses. The statistical analysis plan will be approved during the blinded data review before breaking of the allocation code. Any changes after this signed version will be considered post-hoc.

## Discussion

Iron interventions in early childhood are recommended as an effective intervention to reduce the prevalence of anaemia and improve child development. However, robust evidence for the effects of iron on child development and, importantly, its possible risks is lacking. The BRISC trial will provide definitive evidence for the effects of universal provision of iron, in the form of iron syrup and iron-containing MNPs (the WHO recommendations) on child development, growth, anaemia, and morbidity both immediate and medium term. If effective, it may also establish whether iron supplements or MNPs are better. The results of this rigorous randomised controlled trial will influence global policy guidelines and programmatic practices around universal iron interventions in infants and young children. 

## Data availability

No data are associated with this article.
