# Supplementary material for: The Benefits and Risks of Iron interventionS in Children (BRISC) trial: Statistical analysis plan
Source: F1000Res. 2020 May 22;9:427. [Version 1] doi: 10.12688/f1000research.23383.1 (PMC8984212; doi:10.12688/f1000research.23383.1)
Supplement: Peer review history

# Peer review: Reviewer response for version 1

**DOI:** 10.5256/f1000research.25810.r119673

**Published:** 2022-04-05

**Authors:** Sonia Lewycka

**Affiliations:** 1 Hanoi Unit, Oxford University Clinical Research Unit, Hanoi, Vietnam

This is a very comprehensive and well thought through statistical analysis plan. The main issue that needs clarification is around the timeline. It seems like data has already been collected and possibly analysed. If so, it needs to be made clear that this statistical analysis plan is being published *post hoc*. I have some specific comments below:

1. P1 – introduction – The concern about iron supplementation and infection risk could be elaborated further, though perhaps this is done in the study protocol. Do you mean that iron supplementation might have adverse side-effects? I would say "furthermore" rather than "conversely", and be more specific that there may be adverse effects.
2. P1 – Introduction – I’m not clear on the timeline. In the introduction you say that results are expected to be submitted for publication in late 2020. Have they been submitted yet?
3. P1 – methods – Although it’s too late to address it at this stage, I did wonder why the study team chose 3-months as the supplementation period. This doesn't seem long enough to have a measurable effect on development and growth outcomes. Why not follow WHO recommendation of 6-23 months? Why instead follow up for 9-months without supplementation? Kids may become anaemic again by this time and have lost out on any earlier benefits.
4. P4 – Outcome variables – In the section on additional variables, is there any measure of other dietary intake? Particularly foods rich in vitamin C, which aid iron absorption.
5. P5 – general principles – What do you mean by visit windows? It would also be useful to adjust for baseline measures of outcomes to remove any imbalances due to imperfect randomisation and to increase precision.
6. P8 – multiple testing adjustment – Why no p-values for secondary and exploratory outcomes? I don't think this is necessary as long as you have stated that these are secondary/exploratory outcomes, not adjusted for multiplicity, and should be interpreted with caution. A p-value of <0.001 would be interpreted differently than one of 0.04 even if it is an exploratory analysis. This information is useful for generating hypotheses for testing in future research, even if it can’t provide robust evidence in this study.
7. P8 – efficacy – Three points:
  1) As above, why not adjust for baseline as a covariate rather than assuming a common mean baseline? Or even calculate the difference from baseline to 3 months for each individual and use this as the outcome measure in the regression model? Can then also still be adjusted for baseline.
  2) I don't understand how child can be a random intercept when outcomes at each time-point are included in separate models, so there is only one measurement for each child in each model. If I have misunderstood, this needs to be made clearer.
  3) It would be better to use the same link function for all models, rather than comparing models with different link functions. Will you use GLM for continuous outcomes? Please state this and what link function will be used, since most of your outcomes are continuous.
8. P14 – sensitivity analyses 1b – Why are adjusted models considered as sensitivity analyses? If there is imbalance at baseline and the variables are associated with outcomes, then these could potentially confound the associations, and you would want to include them in your main models. On p15 it states that this was how it was in the original protocol, so it’s not clear why it was changed. There is no need to unblind to see if there is imbalance between arms.
9. P15 – changes from the registry and published study protocol – Has the blinded data review been done yet? Is this version of the statistical analysis plan being published before or after the analyses have been done? If after, then why not publish the final approved version?

Is the study design appropriate for the research question?

Yes

Is the rationale for, and objectives of, the study clearly described?

Yes

Are sufficient details of the methods provided to allow replication by others?

Yes

Are the datasets clearly presented in a useable and accessible format?

Not applicable

Reviewer Expertise:

Epidemiology - design and evaluation of public health interventions

I confirm that I have read this submission and believe that I have an appropriate level of expertise to confirm that it is of an acceptable scientific standard.

# Peer review: Reviewer response for version 1

**DOI:** 10.5256/f1000research.25810.r65657

**Published:** 2020-07-10

**Authors:** Deborah Ridout

**Affiliations:** 1 Population, Policy and Practice Programme, UCL Great Ormond Street Institute of Child Health, University College London, London, UK

This is a well-designed, large randomised trial investigating the use of iron supplementation or home fortification with multiple micronutrient powders, on cognitive development outcome in 8-month old children. The study is set in Bangaldesh and will be conducted over 3 sites. 3,300 children will be recruited and randomised to one of 3 arms, one of which is a placebo. The study is blinded, so that both family and assessor is unaware of group allocation. The study is designed to assess superiority. Secondary outcomes will assess motor and language ability on the Bayley scale as well as prevalence of anaemia and iron deficiency, growth, and infection risks.

 The investigators plan to stratify the randomisation by union and sex, which seems sensible and I wonder if they should also consider weight for age as a stratifying factor.

 An area that may need clarification is around the superiority aspect of the trial, this is mentioned several times, although it is not clear whether the power calculation and planned analyses are considering this design feature.

 There is a possibility that a comparison will be made between the 2 treatments groups, groups 1 & 2, and the plan for this is described well. It is not so clear how these results will be interpreted, is the same effect size of 2 units on the Cognitive Bayley scale appropriate here?

Is the study design appropriate for the research question?

Yes

Is the rationale for, and objectives of, the study clearly described?

Yes

Are sufficient details of the methods provided to allow replication by others?

Yes

Are the datasets clearly presented in a useable and accessible format?

Not applicable

Reviewer Expertise:

Statistician

I confirm that I have read this submission and believe that I have an appropriate level of expertise to confirm that it is of an acceptable scientific standard.

# Response

**Published:** 2020-09-01

**Authors:** Sabine Braat

**Affiliations:** University of Melbourne, Australia

Thank you for the opportunity to respond to the comments of reviewer #1. We appreciate the review provided and have addressed each point below.

*This is a well-designed, large randomised trial investigating the use of iron supplementation or home fortification with multiple micronutrient powders, on cognitive development outcome in 8-month old children. The study is set in Bangaldesh and will be conducted over 3 sites. 3,300 children will be recruited and randomised to one of 3 arms, one of which is a placebo. The study is blinded, so that both family and assessor is unaware of group allocation. The study is designed to assess superiority. Secondary outcomes will assess motor and language ability on the Bayley scale as well as prevalence of anaemia and iron deficiency, growth, and infection risks.*

 Response: We thank the reviewer for the feedback.

*1. The investigators plan to stratify the randomisation by union and sex, which seems sensible and I wonder if they should also consider weight for age as a stratifying factor.*

 Response: We thank the reviewer for this suggestion. The trial is no longer recruiting and finished in 2020. In the “Sensitivity analyses” section we have pre-specified an analysis model that may adjust for characteristics that are unbalanced between treatment arms. This may include weight if it is unbalanced.  

*2. An area that may need clarification is around the superiority aspect of the trial, this is mentioned several times, although it is not clear whether the power calculation and planned analyses are considering this design feature.*

 Response: We thank the reviewer for the comment. In the “Aims” section we refer to the study as a superiority study with the primary objective “...to determine whether 3 months of iron supplementation or home fortification with MNPs is superior to placebo on cognitive development ...”  Both the sample size and the analyses of cognitive development are based on a superiority hypothesis.

*3. There is a possibility that a comparison will be made between the 2 treatments groups, groups 1 & 2, and the plan for this is described well. It is not so clear how these results will be interpreted, is the same effect size of 2 units on the Cognitive Bayley scale appropriate here?*

 Response: We thank the reviewer for this question. The sample size calculation assumed the same effect size for both active treatment groups versus placebo, with 2 units representing a clinically meaningful effect size. The two primary comparisons consist of each active dose vs placebo. When interpreting the results for both active doses, we will take into consideration the findings of the primary outcome (cognitive Bayley scale), secondary outcomes, and safety outcomes.
